# Supplementary material for: Polarity-dependent nonlinear optics of nanowires under electric field
Source: Nat Commun. 2021 Jun 2;12:3286. doi: 10.1038/s41467-021-23488-z (PMC8172856; doi:10.1038/s41467-021-23488-z)
Supplement: Supplementary file 1 — Supplementary Information [file 41467_2021_23488_MOESM1_ESM.pdf]

Supplementary Information

Polarity-dependent nonlinear optics of nanowires under electric field

Regev Ben-Zvi[*1], Omri Bar-Elli[*2], Dan Oron[2✉], Ernesto Joselevich[1✉]

Departments of [†] Materials and Interfaces and [‡] Physics of Complex Systems, Weizmann Institute of Science, Rehovot 76100, Israel. *These authors contributed equally. ✉e-mail: ernesto.joselevich@weizmann.ac.il; dan.oron@weizmann.ac.il

Contents

1. Nanowires growth scheme... 2
2. Additional SEM images for determination of particle location 3
3. Statistics... 4
4. EFM-SHG of a parallel polar nanowire 4
5. Fourier analysis details 5
6. Longitudinal analysis... 8

1. Nanowires growth scheme

The vapor liquid solid growth of surface-guided ZnO nanowires was done in a three-zone furnace in a quartz tube. ZnO powder (99.999% Alfa Aesar) was mixed with graphite powder (99.99% Aldrich) in a 1:1 mass ratio and held at 1050 °C while the substrate was held downstream at 850 °C. The growth was done in a constant flow of 500 sccm N_2 (99.999% Gordon Gas) and a pressure of 400 mbar. In a typical synthesis, a substrate with catalyst was placed on a fused silica carrier plate and inserted to a 25 mm diameter quartz tube. The tube was inserted into a split oven and purged by 4 cycles of pumping to 5 mbar and purging with N_2 at elevated temperature. After purging, N_2 was streamed into the tube, and pressure was maintained at 400 mbar. Once the desired temperature (1050°C) is achieved, the furnace was slide over the sample for one minute for preheating and then slide over the crucible for 20 min for the actual growth. At the end of the growth the furnace is turned off and moved away for the sample to cool down.

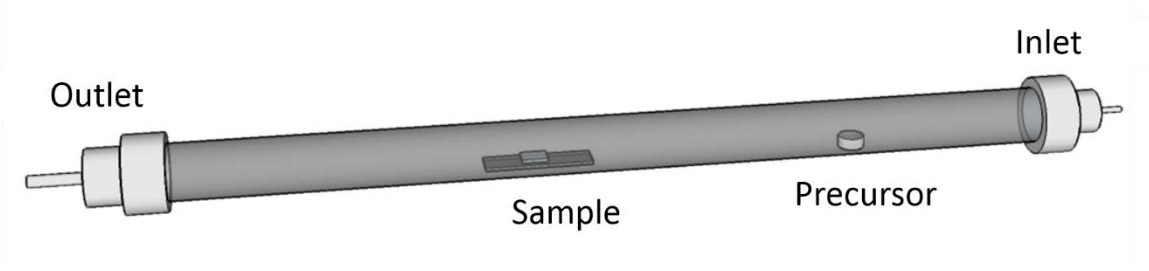

Figure S1. Growth system illustration: A quartz tube placed in a 3 zone furnace. The samples are placed downstream from the precursor.

2. Additional SEM images for determination of particle location

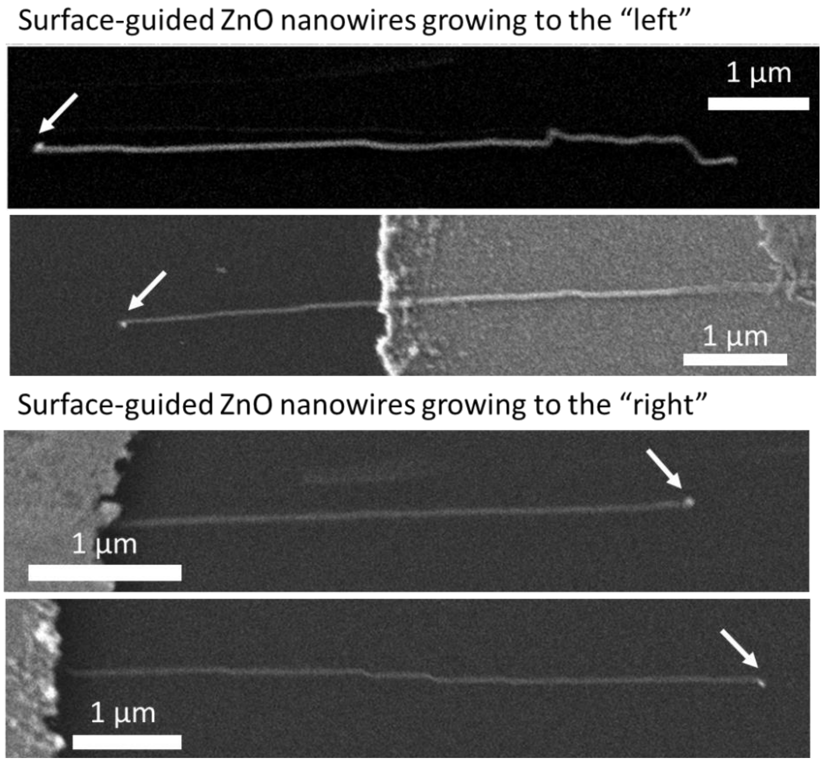

Figure S2. Additional SEM images for determination of particle location. The arrows point to the catalyst nanoparticle at the end of each NWs. The light grey regions on the sample are coated with the Au electrodes.

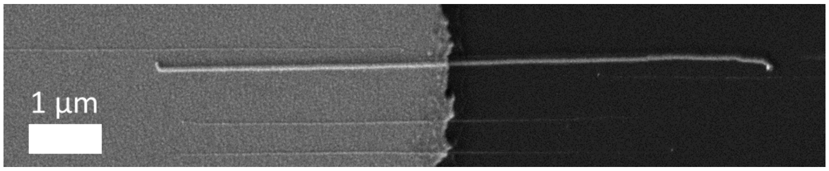

Figure S3. SEM image of a nanowire where the location of the catalyst droplet is ambiguous. According to morphology alone, the two ends of the nanowire look quite similar. Material contrast in SEM imaging allows the detection of the catalyst droplet at the right end of the nanowire. However, since the left part of the nanowire is covered by the metallic electrode, material contrast is obscured and the presence of catalyst droplet cannot be excluded.

3. Statistics

#	Phase (deg)	Growth direction	in-/anti- phase
1	-2.5	Right	in-phase
2	-8	Right	in-phase
3	0	Right	in-phase
4	180	Left	anti-phase
5	180.6	Left	anti-phase
6	180.7	Left	anti-phase
7	179.8	Left	anti-phase
8	-1.5	Right	in-phase
9	-4.5	Right	in-phase
10	1.6	Right	in-phase
11	183.9	Right	anti-phase*
12	177.1	Left	anti-phase
13	181	Left	anti-phase
14	-3.7	Right	in-phase
15	181.9	Left	anti-phase
16	180.3	Left	anti-phase
17	-0.8	Right	in-phase
18	-1.1	Right	in-phase

Table S1. Measured phase and growth direction of 18 different nanowires. *Outlier result. Note that for all the nanowires, the measured phase is either close to 0 deg or to 180 deg within a range of less than ± 5 deg.

4. EFM-SHG of a parallel polar nanowire

In addition to performing EFM-SHG measurements on polar and nonpolar nanowires we examined the polar nanowires along its nonpolar axis. This was achieved by patterning the electrodes parallel to polar nanowires (Figure S4b). Similar to the case of a nonpolar

nanowire, the c-axis is perpendicular to the applied electric field. The difference between these two configurations is the geometric effect, influencing the ratio of SHG signal along different crystal axes. Here too, we expect SH response only at 2ω. However, we observe an additional 1ω component (figure S4c). This can arise from a small misalignment between the nanowire and the electric field. Even the smallest misalignment in electrodes patterning would result in a non-negligible projection of the electric field on the crystal's c-axis, adding a component of the fixed dipole into the SHG response. While the signal at 2ω is comparable to the signal from the other configurations (Figures 3&4 in the main text) the signal at ω is significantly smaller than the signal from perpendicular polar nanowires (Figure 3 of the main text).

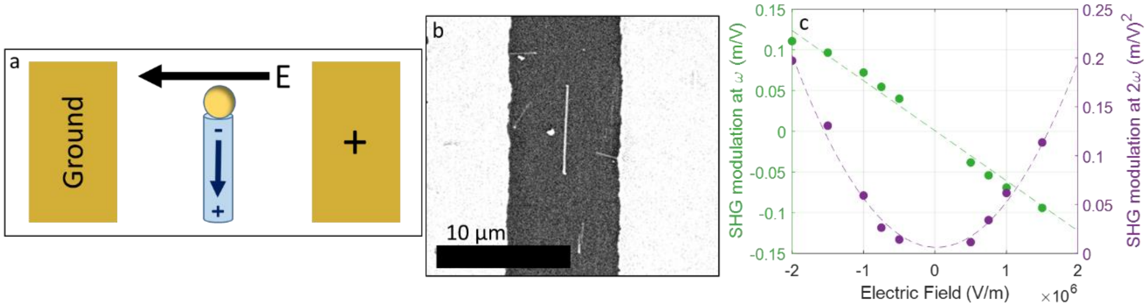

Figure S4. EFM-SHG of a polar nanowire parallel to the electrodes. (a) Schematic representation of the NW (light blue), catalyst (yellow ball), fixed dipole (vertical arrow and -,+ signs), electric field (horizontal arrow) and electrodes (yellow). The c-axis of the ZnO crystal (vertical arrow) is parallel to the nanowire's growth axis and perpendicular to the external electric field (E). (b) SEM image of the device. (c) Amplitude of the signal at 1 kHz (within the noise level) (green) and 2 kHz (purple) from the FFT spectrum as a function of the calculated electric field magnitude. SHG modulation at ω and 2ω is presented in the same scale as in figures 3 and 4.

5. Fourier analysis details

Given here is a more thorough analytic description of the Fourier amplitudes discussed in the main text and presented in figures 3 and 4. Starting with Eq. 4 from the main text where three components contribute to the SHG signal intensity:

$$I(2\omega) \propto |P(2\omega)|^2 = |E^2(\omega)|^2 \left\{ \left[\chi^{(2)}\right]^2 + 2\chi^{(2)}\chi^{(3)}E_{AC} + \left[\chi^{(3)}\right]^2 |E_{AC}(\omega_{AC})|^2 \right\}$$

Explicitly express the external field as a sine wave with amplitude E_0:

$$E_{AC}(\omega_{AC}) = E_0 \sin(\omega_{AC} t)$$

Express the oscillating terms in positive and negative frequencies:

$$\begin{aligned}
&\left|P(2\omega)\right|^2 = \left|E^2(\omega)\right|^2 \left\{\left[\chi^{(2)}\right]^2 + 2\chi^{(2)}\chi^{(3)}E_0\sin(\omega_{AC}t) + \left[\chi^{(3)}\right]^2 \left|E_0\sin(\omega_{AC}t)\right|^2\right\} = \\
&= \left|E^2(\omega)\right|^2 \left\{\left[\chi^{(2)}\right]^2 + 2\chi^{(2)}\chi^{(3)}E_0\sin(\omega_{AC}t) + \left[\chi^{(3)}\right]^2 \frac{E_0^2}{2}\left(1-\cos(2\omega_{AC}t)\right)\right\} = \\
&= \left|E^2(\omega)\right|^2 \left\{\underbrace{\left(\left[\chi^{(2)}\right]^2 + \left[\chi^{(3)}\right]^2 \frac{E_0^2}{2}\right)}_{0Hz} + \underbrace{2\chi^{(2)}\chi^{(3)}E_0\sin(\omega_{AC}t)}_{1\omega_{AC}} - \underbrace{\left[\chi^{(3)}\right]^2 \frac{E_0^2}{2}\left(\cos(2\omega_{AC}t)\right)}_{2\omega_{AC}}\right\} = \\
&= \left|E^2(\omega)\right|^2 \left\{\left(\left[\chi^{(2)}\right]^2 + \left[\chi^{(3)}\right]^2 \frac{E_0^2}{2}\right) + 2\chi^{(2)}\chi^{(3)}E_0 \frac{e^{i\omega t} - e^{-i\omega t}}{2i} - \left[\chi^{(3)}\right]^2 \frac{E_0^2}{2}\frac{e^{i2\omega t} + e^{-i2\omega t}}{2}\right\} = \\
&= \left|E^2(\omega)\right|^2 \left\{\left(\left[\chi^{(2)}\right]^2 + \left[\chi^{(3)}\right]^2 \frac{E_0^2}{2}\right) + \underbrace{\left[2\chi^{(2)}\chi^{(3)}E_0 \frac{e^{i\omega t}}{2i} - \left[\chi^{(3)}\right]^2 \frac{E_0^2}{2}\frac{e^{i2\omega t}}{2}\right]}_{\text{positive frequencies}} - \underbrace{\left[2\chi^{(2)}\chi^{(3)}E_0 \frac{e^{-i\omega t}}{2i} + \left[\chi^{(3)}\right]^2 \frac{E_0^2}{2}\frac{e^{-i2\omega t}}{2}\right]}_{\text{negative frequencies}}\right\} = \\
&= \left|E^2(\omega)\right|^2 \left\{\underbrace{\left(\left[\chi^{(2)}\right]^2 + \left[\chi^{(3)}\right]^2 \frac{E_0^2}{2}\right)}_{\hat{a}_{0Hz}} - \left[i\underbrace{\chi^{(2)}\chi^{(3)}E_0}_{\hat{a}_{1\omega}} e^{i\omega t} + \underbrace{\frac{1}{4}\left[\chi^{(3)}\right]^2 E_0^2}_{\hat{a}_{2\omega}} e^{i2\omega t}\right] - \left[i\chi^{(2)}\chi^{(3)}E_0 e^{-i\omega t} + \frac{1}{4}\left[\chi^{(3)}\right]^2 E_0^2 e^{-i2\omega t}\right]\right\}
\end{aligned}$$

From here, we can directly write the absolute value of the Fourier amplitudes at 0Hz, ω_{AC} and, $2\omega_{AC}$ by dropping the sign and phase of the terms marked in the last line:

$$\begin{aligned}
\hat{a}_{0Hz} &= \left[\chi^{(2)}\right]^2 + \frac{1}{2}\left[\chi^{(3)}\right]^2 E_0^2 \\
\hat{a}_{1\omega} &= \chi^{(2)}\chi^{(3)}E_0 \\
\hat{a}_{2\omega} &= \frac{1}{4}\left[\chi^{(3)}\right]^2 E_0^2
\end{aligned}$$

Note the contribution of $\chi^{(3)}$ to the DC (0Hz) amplitude, this contribution depends on the square of the magnitude of the external field and is exactly double the Fourier amplitude $\hat{a}_{2\omega}$. At low applied voltage this contribution is negligible as is evident by the lack of signal at $2\omega_{AC}$. Additionally, the phase of the linear component is $\pm i$ depending on the sign (coordinate in the lab frame) of our applied external field (E_0). A constant phase in Fourier

space is meaningless here; rather the difference between the two options (180°) is of significance, as shown in figure 2 of the main text. The normalized amplitudes presented in figures 3b, 3c, and 4d of the main text are given by:

$$a_{1\omega} = \frac{\hat{a}_{1\omega}}{\hat{a}_{0Hz}} = \frac{\chi^{(2)}\chi^{(3)}E_0}{\left[\chi^{(2)}\right]^2 + \frac{1}{2}\left[\chi^{(3)}\right]^2 E_0^2}$$

$$a_{2\omega} = \frac{\hat{a}_{2\omega}}{\hat{a}_{0Hz}} = \frac{\frac{1}{4}\left[\chi^{(3)}\right]^2 E_0^2}{\left[\chi^{(2)}\right]^2 + \frac{1}{2}\left[\chi^{(3)}\right]^2 E_0^2}$$

We define a single fitting parameter for each expression: $r = \frac{\chi^{(3)}}{\chi^{(2)}}$ and $r' = \left[\frac{\chi^{(3)}}{\chi^{(2)}}\right]^2$ respectively.

$$a_{1\omega} = \frac{rE_0}{1+\frac{1}{2}r^2E_0^2} \quad ; \quad a_{2\omega} = \frac{\frac{1}{4}r'E_0^2}{1+\frac{1}{2}r'E_0^2}$$

Using the above expressions we perform a fit to extract the ratio between $\chi^{(2)}_{zzz}$ and $\chi^{(3)}_{zzzz}$.

#	r	$\sqrt{r'}$
5	3.5e-09	
6	9.5e-09	
7	2.9e-09	1.3e-08
8	2.3e-09	1.6e-08
13	1.3e-08	5.8e-08
16	3.4e-09	

Table S2. Summary of fit results for the ratio of the nonlinear coefficients. Nanowire # follows from the previous table.

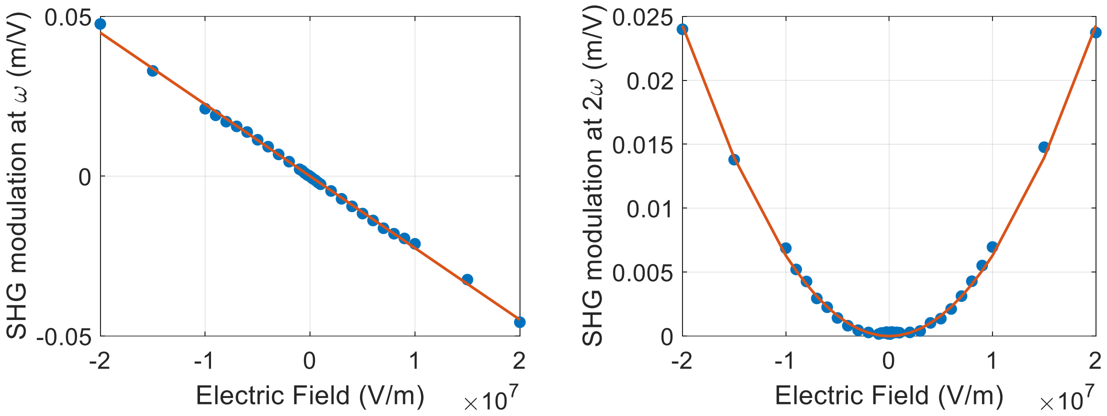

Figure S5. Examples of fits used to extract the ratio between the nonlinear coefficients.

The median of the fit results for *r* was used to present the estimated values in the main text. The spread of the results $(2.3 - 13 \cdot 10^{-9})$ is most likely due to difficulties in estimating the exact amplitude of the applied electric field. The small thickness of the gold electrodes (few 100s of nm) makes them different from an ideal capacitor, an effect we neglect in the calculations. Furthermore, the local environment may include features around the nanowire (metallic or dielectric) left over from the photolithography process that may affect the local electric field differently in each experiment. An error in the field amplitude would affect the ratio *r'* more than the ratio *r* due to their quadratic and linear dependence respectively.

6. Longitudinal analysis

We performed an EFM-SHG scan along a single polar nanowire. It appears that around the center of the nanowire there is a smaller modulation of the SHG signal. This is most likely due to the deviation of the electrodes from an ideal capacitor thus a non-uniform electric field is present along the nanowire.

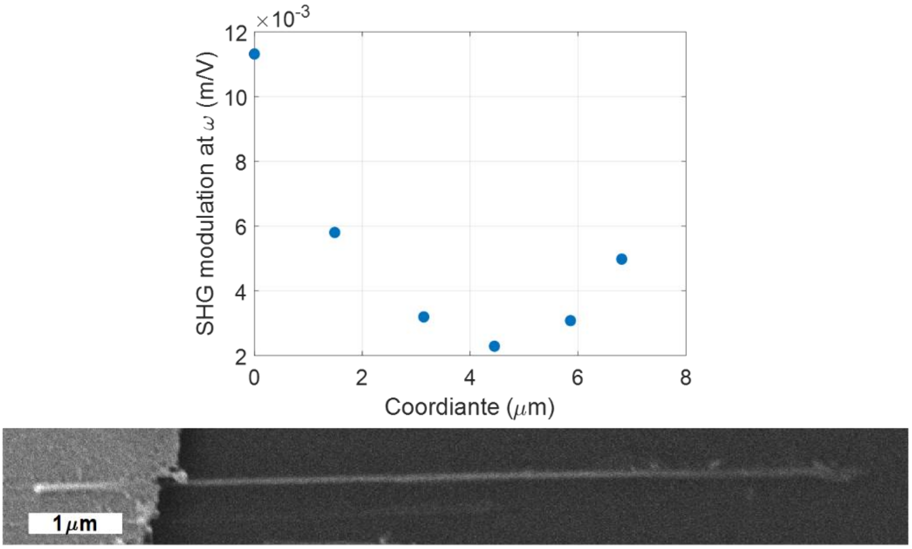

Figure S6. SHG modulation from different locations along a single nanowire. The nanowire's coordinate goes from left (close to the left electrode) to right.
